# T cell repertoire in peripheral blood as a potential biomarker for predicting response to concurrent cetuximab and nivolumab in head and neck squamous cell carcinoma

**DOI:** 10.1136/jitc-2022-004512

**Published:** 2022-06-08

**Authors:** Xuefeng Wang, Jameel Muzaffar, Kedar Kirtane, Feifei Song, Matthew Johnson, Michael J Schell, Jiannong Li, Sean J Yoder, Jose R Conejo-Garcia, Jose A Guevara-Patino, Marcelo Bonomi, Priyanka Bhateja, James W Rocco, Conor E Steuer, Nabil F Saba, Christine H Chung

**Affiliations:** 1 Department of Biostatistics and Bioinformatics, H Lee Moffitt Cancer Center and Research Center Inc, Tampa, Florida, USA; 2 Department of Head and Neck-Endocrine Oncology, H Lee Moffitt Cancer Center and Research Center Inc, Tampa, Florida, USA; 3 Department of Molecular Genomics Core, H Lee Moffitt Cancer Center and Research Center Inc, Tampa, Florida, USA; 4 Department of Immunology, H Lee Moffitt Cancer Center and Research Center Inc, Tampa, Florida, USA; 5 Department of Internal Medicine, The Ohio State University Wexner Medical Center, Columbus, Ohio, USA; 6 Department of Otolaryngology, The Ohio State University Wexner Medical Center, Columbus, Ohio, USA; 7 Department of Hematology and Medical Oncology, Winship Cancer Institute, Emory University, Atlanta, Georgia, USA

**Keywords:** head and neck neoplasms, immunotherapy

## Abstract

**Background:**

T cell receptor (TCR) signaling profile is a fundamental property that underpins both adaptive and innate immunity in the host. Despite its potential clinical relevance, the TCR repertoire in peripheral blood has not been thoroughly explored for its value as an immunotherapy efficacy biomarker in head and neck squamous cell carcinoma (HNSCC). The purpose of the present study is to characterize and compare the TCR repertoire in peripheral blood mononuclear cells (PBMC) from patients with HNSCC treated with the combination of cetuximab and nivolumab.

**Methods:**

We used the immunoSEQ assay to sequence the TCR beta (TCR-B) chain repertoire from serially obtained PBMC at baseline and during the treatments from a total of 41 patients who received the combination (NCT03370276). Key TCR repertoire metrics, including diversity and clonality, were calculated and compared between patients with different therapy responses and clinical characteristics (eg, human papillomavirus (HPV) status and smoking history). Patient survival outcomes were compared according to patient groups stratified by the TCR-B clonotyping. To confirm the observed patterns in TCR spectrum, samples from patients who achieved complete response (CR) and partial response (PR) were further profiled with the immunoSEQ deep resolution assay.

**Results:**

Our data indicated that the patients who achieved CR and PR had an increased TCR sequence diversity in their baseline samples, this tendency being more pronounced in HPV-negative patients or those with a smoking history. Notably, the CR/PR group had the lowest proportion of patients with oligoclonal TCR clones (2 out of 8 patients), followed by the stable disease group (9 out of 20 patients) and lastly the progressive disease group (7 out of 10 patients). An overall trend toward favorable patient survival was also observed in the polyclonal group. Finally, we reported the shared TCR clones across patients within the same response group, as well as the shared clones by aligning immunoSEQ reads with TCR data retrieved from The Cancer Genome Atlas- head and neck squamous cell carcinoma (TCGA-HNSC) cohort.

**Conclusions:**

Our data suggest that, despite the great clinical heterogeneity of HNSCC and the limited responders in the present cohort, the peripheral TCR repertoires from pretreatment PBMC may be developed as biomarkers for the benefit of immunotherapy in HNSCC.

**What is already known on this topic**

- T cell receptor (TCR) repertoire in tumor and peripheral blood provides fundamental information for understanding T cell dynamics in response to cancer and its treatments.
- Few markers have been developed for predicting the response to cetuximab and nivolumab combination therapy for patients with recurrent and/or metastatic head and neck squamous cell carcinoma (HNSCC).
- We hypothesized that patients with HNSCC who achieved the same response to concurrent cetuximab and nivolumab might share similar TCR repertoire patterns in pre-treatment peripheral blood, representing a unique pre-existing immune state.

**What this study adds**

- Based on TCR-beta repertoire sequencing, our study demonstrated the prognostic potential for TCR repertoire clonotyping in pretreatment peripheral blood mononuclear cells to predict therapy response and survival outcomes for patients with HNSCC treated with the combination therapy.
- The reliability of the proposed top–down clonotyping approach was further validated by results from DeepTCR sequencing on a subset of samples.
- In addition, our data revealed distinct peripheral TCR repertoire clonality/diversity among smoking and HPV patient subgroups.
- Clonotype trajectory and clustering analyses were also performed to track the shared complementarity-determining region 3 sequences across time points and among patients with HNSCC.

**How this study might affect research, practice, or policy**

- The findings presented here provide important pilot evidence on using peripheral blood TCR repertoires as a non-invasive and repeatable approach for predicting and tracking the host immune responses that are signaling the benefit of immunotherapy in HNSCC.

## Introduction

Head and neck squamous cell carcinoma (HNSCC) is one of the most prevalent cancers in the USA and around the world.^1^ The 5-year overall survival rate of patients with HNSCC after diagnosis is less than 50%, and those with human papillomavirus (HPV)-negative tumors have an even worse prognosis. Despite advances in surgery, radiotherapy, and chemotherapy, patients with recurrent and/or metastatic (R/M) HNSCC have seen minimal or no improvements in survival over recent decades. On the other hand, HNSCC is known to be immunogenic. Recent studies have demonstrated the beneficial effects of cancer immunotherapy using immune checkpoint blockades (ICBs) in treating a subgroup of patients with HNSCC.^2 3^ However, the response rate remains low, with less than 20% of patients achieving durable response in the anti-programed cell death 1 (PD-1) ICB. As a result, there is an urgent need to develop predictive biomarkers for immunotherapy to select targeted patients for more precise treatments and clinical trials.

Characterization of T cell receptor (TCR) repertoire in peripheral blood and tissue samples offers a promising and highly informative source for predicting and monitoring both host-tumor and antitumor immune conditions. TCR profiling approaches based on next-generation sequencing allows for targeted high-throughput investigation of the complementarity-determining region 3 (CDR3), the most variable part of TCR chains. Each CDR3 sequence acts as a unique label or barcode that can be used to track T cell composition, clonal lineage, repertoire dynamics and diversity across treatment phases or among patient groups. Previous studies have demonstrated that the diversity of the baseline TCR of tumor infiltrating lymphocytes (TILs) are predictive to patient survival and clinical outcomes in multiple cancers with conventional treatments and immunotherapies.^4–7^ Although TCR phenotypes in peripheral blood can be affected by various clinical factors, the blood-based repertoire analysis is more appealing for tracking the host immune response than tissue-based analysis due to the ease in sample collection. Multiple recent studies in several cancer types, such as melanoma, have demonstrated how the TCR phenotyping of peripheral blood mononuclear cells (PBMCs) can be relevant (alone or in combination with other biomarkers) in predicting the ICB effectiveness.^8–10^ The richness of TCR sequences was also found to be favorably associated with overall treatment response in patients with head and neck cancer treated with cetuximab, an epidermal growth factor receptor-targeted therapy.^11^ To the best of our knowledge, no study has yet systematically investigated the prognostic value of TCR repertoires in peripheral blood for predicting immunotherapy efficacy (alone or in combination) in HNSCC.

In this study, we sequenced the CDR3 region of the TCR beta (TCR-B) chain of PBMC samples from patients with HNSCC treated with combination of cetuximab and nivolumab using the immunoSEQ assay platform. The main goal is to examine whether the baseline TCR repertoire metrics (such as diversity, gene usage and clonal abundance) obtained from pretreatment blood samples can be used to predict which patients will benefit from the combination immunotherapy. To study TCR clonal dynamics and selection pressure imposed by the treatment, we also compared the matched TCR repertoire profiled at different clinical events. Overall, our findings imply that the blood based TCR-beta clonality may serve as prognostic biomarkers in HNSCC treated with the combined therapy.

## Material and methods

### Patient sample collection

**Figure 1:**
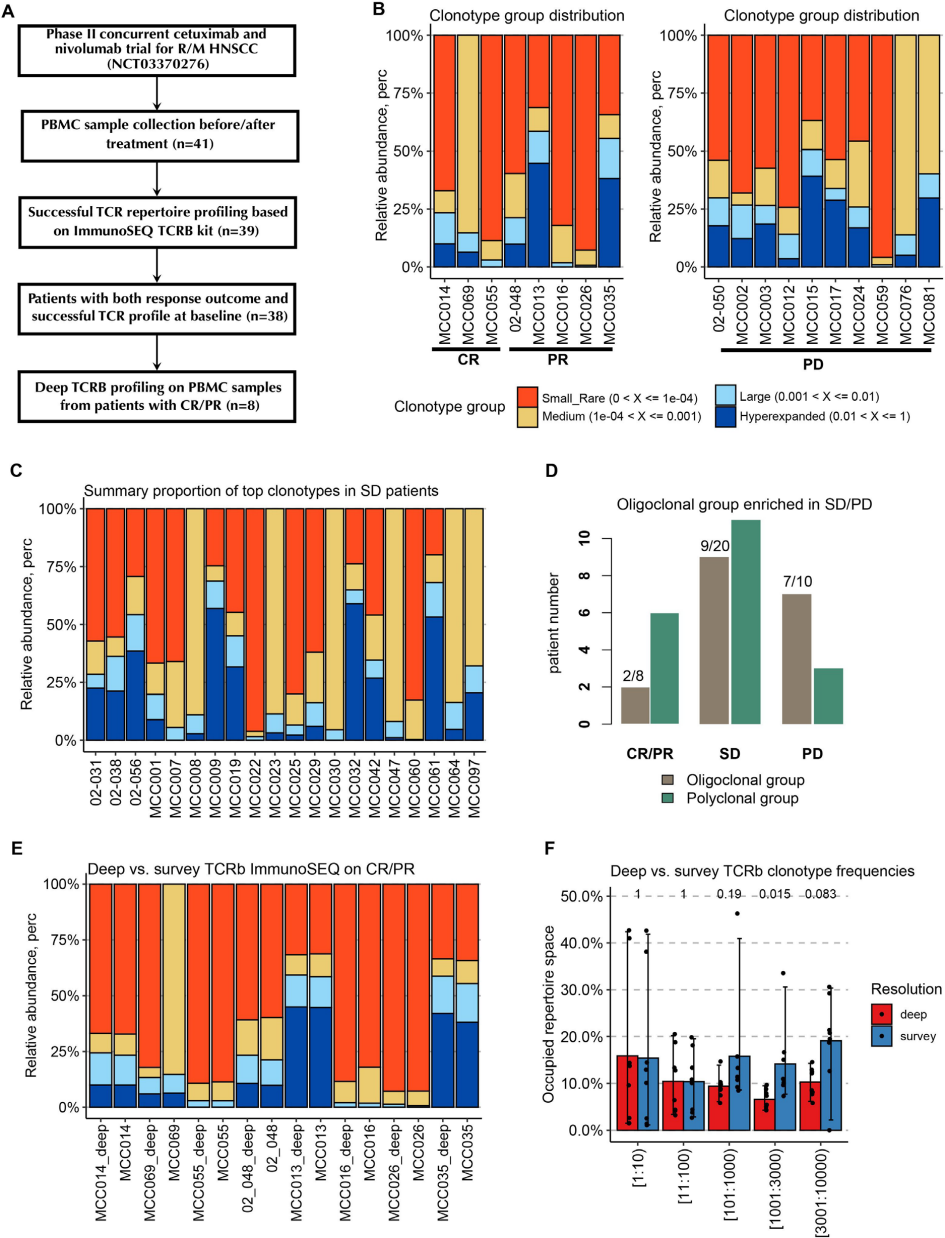
Patients with CR/PR have a less oligoclonal peripheral TCR expansion (higher TCR diversity) prior to the combination therapy. (A) Flowchart of PBMC sample selection for the survey and deep immunoSEQ TCR-B assay. (B) Distribution of four clonotype groups in the CR and PR patient group (defined based on relative abundance of the clone sequences). (C) Distribution of four clonotype groups in the PD patient group. (D) The proportion of patients with oligoclonal (the combined proportion of large and hyperexpanded clonotypes >25%) sequences in each response group. (E) Comparison of clonotype spectrums estimated from the survey and the follow-up DeepTCR-B sequencing based on matched CR/PR baseline PBMC samples. (F) Comparison of estimated clonotype frequencies of top clonotype groups from the survey and the follow-up deep and TCR-B sequencing. CR, complete response; HNSCC, head and neck squamous cell carcinoma; PBMC, peripheral blood mononuclear cells; PD, progressive disease; PR, partial response; SD, stable disease; TCR-B, TCR beta; TCR, T cell receptor.

All samples were collected from patients enrolled in a phase I/II study of concurrent cetuximab and nivolumab in patients with R/M HNSCC (NCT03370276), and the detailed study results have been published.^12 13^ Briefly, patients were recruited across three sites: Moffitt Cancer Center, Emory University, and the Ohio State University. The clinical events include pre-therapy (screening), cetuximab lead-in 2 weeks before Cycle 1 Day 1 of cetuximab and nivolumab (C1D1), Cycle 4 D1 (C4D1), end of treatment. The baseline clinical and demographic characteristics are shown in table 1. The flowchart of study patients is depicted in figure 1A. Briefly, the PBMC from 41 patients were analyzed for TCR repertoire. HNSCC disease sites include 13 oral cavity, 23 oropharynx, 3 larynx, 1 hypopharynx and 1 from unknown site. Twenty-three patients (56%) were HPV-positive based on the p16 immunohistochemistry. PBMC samples from a total of 38 patients were included in the final analysis, including 8 patients with complete response (CR) or partial response (PR), 20 with stable disease (SD), and 10 with progressive disease (PD). Patient’s blood samples were collected into a standard 10 ml K^2^ EDTA Vacutainer tube (BD. 366433) or LBgard Blood Tubes (Biomatrica, REF: 68 021–001). The EDTA-tube collected blood samples were transferred into 50 mL conical Falcon tube, and then centrifuged via density-gradient separation at room temperature to separate out the PBMC and the cell pellet. Then the PBMC were isolated from an aliquot of the buffy coats via density-gradient separation and were either used fresh or cryopreserved in freezing media (80% fetal bovine serum supplemented, 6% RPMI (Roswell Park Memorial Institute) media with 14% DMSO (dimethyl sulfoxide)) until DNA extraction. The LBgard Tube-collected blood samples were centrifuged at 3000 g, 15 min at room temperature to separate out the plasma, the buffy coat and red blood cell layer. The buffy coats were frozen in −80°C until DNA extraction. Genomic DNA was extracted from each sample using the QIAamp DNA blood mini kit (Qiagen, Hilden, Germany) according to the manufacturer’s instructions. DNA concentration and quality were measured using NanoDrop spectrophotometer (NanoDrop Technologies, Wilmington, Delaware, USA) and Invitrogen Qubit dsDNA HS and BR Assay Kits (Thermo Fisher Scientific, REF: Q32853, Thermo Scientific, Waltham, Massachusetts, USA). PBMC, buffy coat processing and DNA isolation and quantification were performed at the Moffitt Cancer Center Tissue Core.

**Table 1:** Clinical characteristics of 41 patients with head and neck squamous cell carcinoma (received concurrent cetuximab and nivolumab) profiled with peripheral blood mononuclear cells immunosequencing

	Overall (N=41)
Age on study	
Mean (SD)	61.6 (11.3)
Median (min, max)	64.0 (24.0, 78.0)
Gender	
Female	8 (19.5%)
Male	33 (80.5%)
Race	
Asian	1 (2.4%)
Black or African American	2 (4.9%)
White	37 (90.2%)
Unknown	1 (2.4%)
Primary site	
Hypopharynx	1 (2.4%)
Larynx	3 (7.3%)
Oral cavity	13 (31.7%)
Oropharynx	23 (56.1%)
Unknown primary	1 (2.4%)
p16 status	
Negative	18 (43.9%)
Positive	23 (56.1%)
Smoking history	
Current smoker	4 (9.8%)
Previous smoker	21 (51.2%)
Never smoked	16 (39.0%)
ECOG PS	
0	14 (34.1%)
1	25 (61.0%)
2	2 (4.9%)
Prior immunotherapy	
No	24 (58.5%)
Yes	17 (41.5%)
Best response	
Complete response	3 (7.3%)
Partial response	6 (14.6%)
Stable disease	20 (48.8%)
Progressive disease	10 (24.4%)
Not assessed	2 (4.9%)
Survival status	
Alive	14 (34.1%)
Death	27 (65.9%)

ECOG PS, Eastern Cooperative Oncology Group Performance Status.

### ImmunoSEQ assay

Survey resolution TCR repertoire analysis was carried out with the use of the Adaptive Biotechnologies immunoSEQ assay, which employs bias-controlled multiplex PCR amplification and high-throughput sequencing to target rearranged TCR genes. As a follow-up experiment, all PBMC samples from eight patients who achieved CR/PR were further profiled with deep resolution immunoSEQ assay (DeepTCR). Briefly, the manufacturer’s protocol was followed in order to utilize the immunoSEQ hsTCR-B kit to amplify the CDR3 locus from 1 microgram of genomic DNA extracted from PBMCs. Following the confirmation of amplification and successful final library preparation, sequencing was performed on the Illumina NextSeq 500 to a depth of approximately 2 million sequencing reads per sample. The data were then analyzed using the Adaptive Biotechnologies ImmunoSEQ Analyzer software (V.3.0), which identifies the V, D, and J genes, filters non-productive sequences, and reports and tracks T cell clonality. All TCR-sequencing were performed in Moffitt Cancer Center shared resource facility Molecular Genomics Core.

### TCR repertoire and statistical analyses

The preliminary analysis results, quality control, and summary statistics of CDR3 sequences (templates) were performed using the online ImmunoSEQ Analyzer portal provided by Adaptive Biotechnologies (http://www.immunoseq.com). All samples were quantified for the absolute total rearrangements, which is the sum of unique detectable CDR3 sequences (or rearrangements) in the TCR-B chain. To evaluate the clonality of T cell repertoire, we utilized the Simpson Clonality Index, calculated as the square root of Simpson diversity metric. Simpson Clonality is generally recommended over Shannon Clonality (another commonly used diversity score) because it is less sensitive to differences in total number of sequences. All Simpson Clonality scores were summarized based on productive rearrangements, that is, CDR3 sequences that are capable of producing a functional peptide. All statistical analyses were conducted in R (V.4.0.2). We assessed the differences of total rearrangements and clonality among different groups using the non-parametric Wilcoxon signed-rank tests (with two groups) and Kruskal-Wallis test (to compare more than two groups). Specifically, we compared the TCR diversity metrics among different therapy-response groups, groups based on smoking history, as well as patient groups stratified by HPV status. All statistical tests were two-sided and p value ≤0.05 was considered statistically significant. In order to prevent the possible bias from a single diversity metric selected, we further explored the repertoire differences among various patient subgroups using Hill numbers (with diversity orders between 1 and 5). Hill numbers are a mathematically unified family of diversity indices differing among different diversity order, or Q values. For example, Hill numbers equal to repertoire richness (ie, total rearrangements) when Q=0, and is equivalent to Shannon entropy when Q=1. Based on the frequencies (X) of detectable read, we classified all CDR3 sequences into four clonotype groups: Hyperexpanded (when X>0.01), large (0.001<X≤0.01), medium (10^–4^ <X≤0.001), and small/rare group (X<10^–4^). In this study, we further define the patients having >25% of total reads dominated by large or hyperexpanded (TCR-B) clones as oligoclonal group; and <25% as polyclonal group. We compared the overall survival (OS) between the polyclonal and oligoclonal groups using the Kaplan-Meier analysis and log-rank tests, as well as the Cox regression model adjusting for clinical covariates.

The additional clonal diversity metrics and clonal proportions were calculated using the functions repDiversity and repClonality in the R package ‘immunarch’^14^ (V.0.6.6), respectively. TCR repertoires were further analyzed for the usage of TRBV and TRBJ genes complied in the IMGT database, which is quantified as relative frequencies of shared V/J gene segments flanking all the CDR3 sequences in one sample. Ambiguous gene assignments were excluded in calculating the gene usage. For each sample, the spectrum of CDR3 length and its association with gene usage were examined using the CDR3 spectratyping plot. The gene usage patterns across patients were explored and visualized using the unsupervised clustering methods (K-means and hierarchical clustering) and Jensen-Shannon (JS) divergence, based on the geneUsageAnalysis function as implemented in ‘immunarch’. The trackClonotypes function, also included in ‘immunarch’, was used to track and annotate TCR clonotypes across time points. Finally, to discover the shared clones across different patients, we clustered all TCR-B amino acid sequences using the software GIANA, which implements a computationally efficient clustering framework based on isometric transformation of CDR3 sequences.^15^

To align TCR CDR3 clones with those obtained from tumor samples in this study, we assembled an HNSCC-specific reference library by retrieving TCR sequences from all tumor RNA sequencing (RNAseq) data available in the The Cancer Genome Atlas-head and neck squamous cell carcinoma (TCGA-HNSC) cohort. RNAseq BAM files from a total of 502 HNSCC tumor samples were downloaded from the TCGA GDC data portal (https://portal.gdc.cancer.gov), which were subsequently converted to FASTQ files using bedtools.^16^ The software MiXCR^17^ (V.3.0.13) was used to extract and quantify TCR CDR3 sequences from FASTQ files following the recommended paired-end RNAseq processing workflow.

## Results

### Baseline TCR-B clonality correlate with clinical and therapy outcomes

First, we examined the effects of pre-existing TCR repertoire characteristics from baseline PBMC on patient outcomes to the combination therapy. For the patient response outcomes, we classified patients into three groups based on the best response, that is, CR/PR, SD, and PD. Out of 41 patients studied, 38 had both response data and baseline TCR-sequencing data available. As shown in figure 1B, C, we evaluated the clonotype spectrum in each patient response group based on the relative abundance of four clonotype groups. The clonality spectrum revealed that the large and hyperexpanded clonotypes (with the relative abundance >0.001) were more enriched in the PD group. There are no patients in the CR group, and only two patients in the PR group (MCC013 and MCC035) having the combined proportion of the expanded clonotypes greater than 25% (ie, the oligoclonal patient). The proportions of oligoclonal patient in the CR/PR, SD and PD groups are 25% (2/8), 45% (9/20), and 70% (7/10), respectively, as shown in figure 1D. Accordingly, the steadily increased proportions indicate that patients with polyclonal TCR repertoire in the baseline PBMC are more likely to benefit from the cetuximab and nivolumab combination therapy. To assess the reproducibility of the proposed clonotyping strategy and the resulting conclusion, we compared the summary proportions of the clonotypes calculated based on the follow-up DeepTCR sequencing (applied to the matched samples from eight CR/RP patients). As illustrated in figure 1E, overall DeepTCR profiles generated clonotype abundance spectrums that are highly consistent with the survey resolution data. Only the pretreatment sample from MCCC069 demonstrated an outlier distribution in the medium and small/rare clonotype groups, owing to the exceptionally low productive templates (<2000 total rearrangements) yielded from this sample. Figure 1F confirms this point further by showing the increased discrepancy in estimating frequencies of rarer clonotypes. This phenomenon verifies the main translational advantage of the top–down clonotyping approach—it is remarkably consistent across sequencing depth and input DNA quantity.

**Figure 2:**
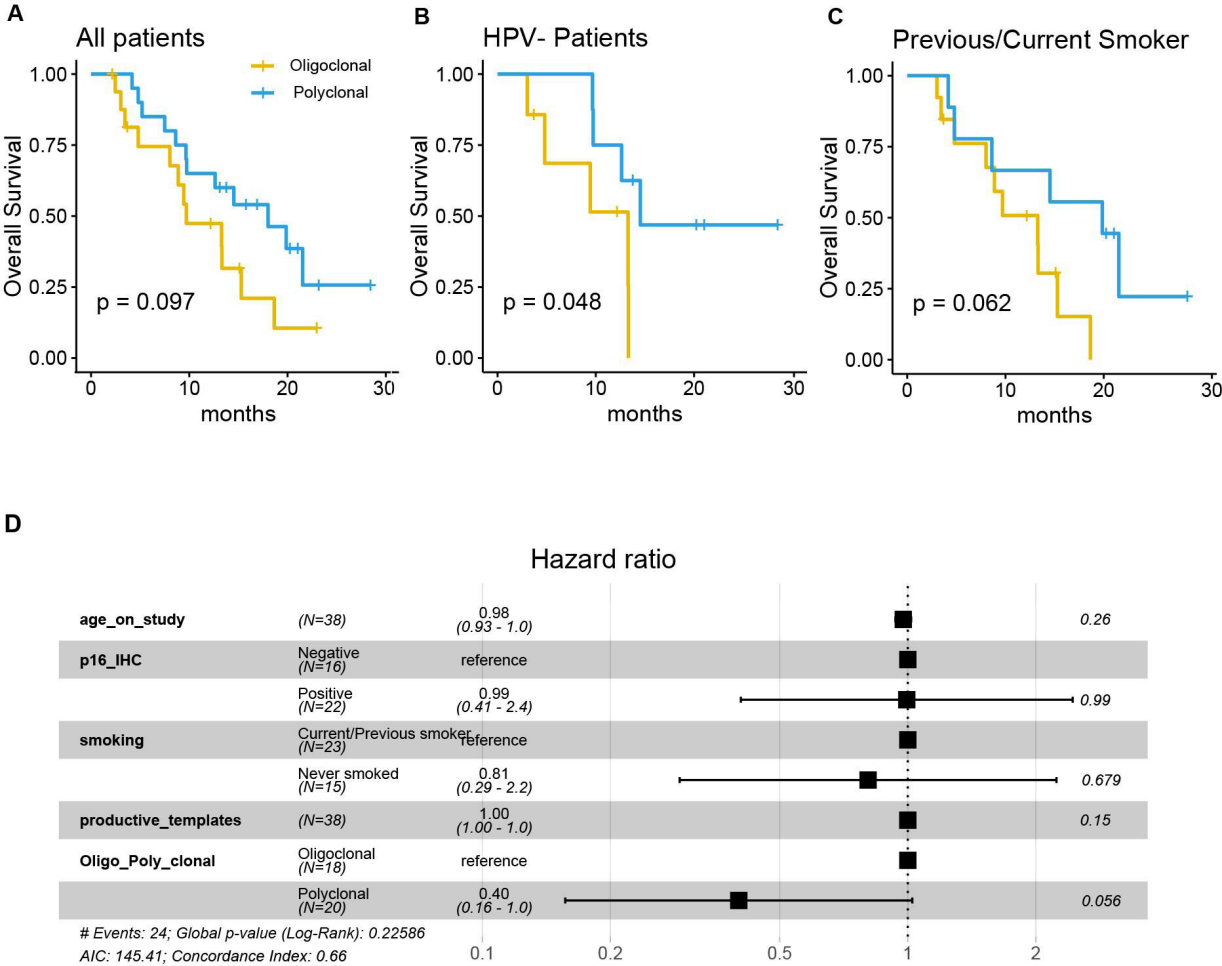
The pretreatment peripheral TCR clonotyping is prognostic for overall survival. (A–C) Kaplan-Meier survival curves comparing patients with oligoclonal and polyclonal TCR repertories in (A) all patients; (B) HPV-negative patients and (C) who are ever smokers. (D) Summary of results from the Cox proportional hazards regression model (to test the association with clonotyping and overall survival) by adjusting age, smoking, productive templates. HPV, human papillomavirus; IHC, immunohistochemistry; TCR, T cell receptor.

When comparing the OS of the two clonotype-based patient groups, the polyclonal patients likewise showed a favorable prognosis (log-rank test, p=0.097), as shown in figure 2. Similar to the TCR diversity findings, we discovered that the improvement in OS became significant when only considering the HPV-negative patients (p=0.048) and approached borderline statistical significance in patients with smoking history (p=0.062) (for HPV-positive and never smokers, refer to online supplemental figure S1). After adjusting for covariates including age, HPV status, smoking and the total productive templates in the multivariable Cox regression model, the association between the clonotype-based patient group and OS approached marginal statistical significance (p=0.056), as illustrated in figure 2D.

10.1136/jitc-2022-004512.supp1 Supplementary data

**Figure 3:**
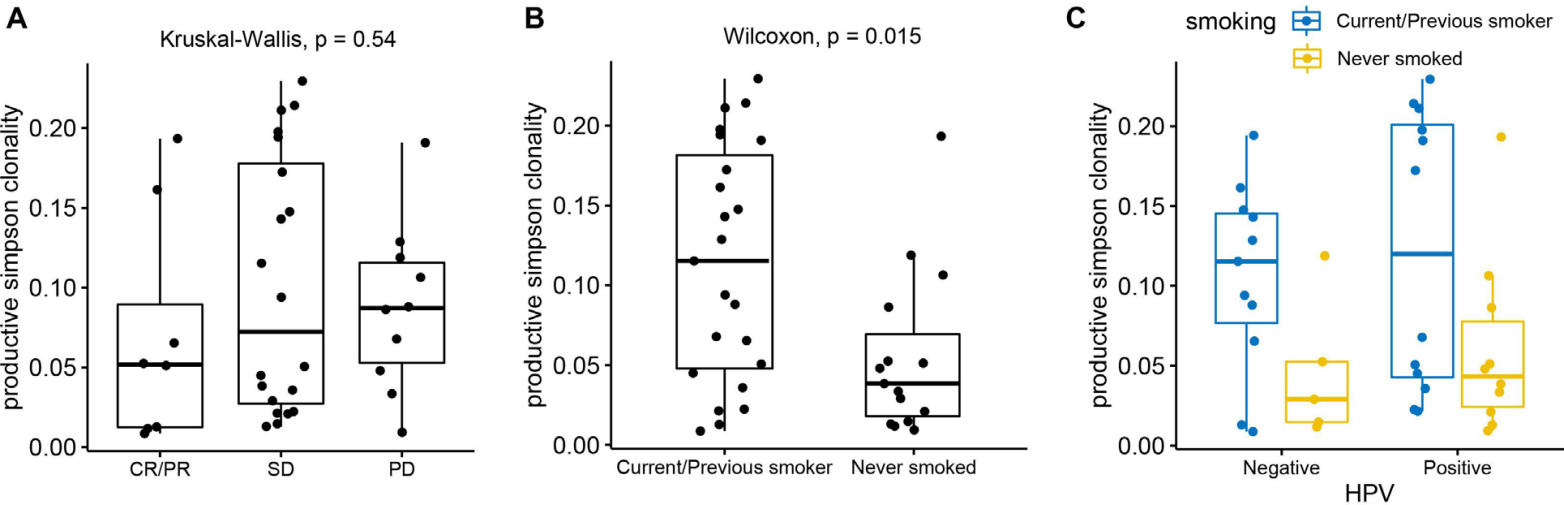
Association between TCR repertoire diversity in baseline PBMC and clinical outcomes of patients with HNSCC. (A) The CR/PR patients have the lowest productive Simpson Clonality score, followed by SD and PD. (B) The never smoked patients with HNSCC show significantly lower productive Simpson clonality (p=0.015) in contrast to ever smokers. (C) The further stratification by HPV status shows the similar pattern of diversity discrepancy driven by smoking status. CR, complete response; HNSCC, head and neck squamous cell carcinoma; HPV, human papillomavirus; PBMC, peripheral blood mononuclear cells; PD, progressive disease; PR, partial response; SD, stable disease; TCR, T cell receptor.

To further examine how the blood TCR repertoire at baseline is associated with patient clinical and treatment outcomes, we compared TCR richness (measured by total rearrangements) and diversity metrics (measured by Simpson Clonality) across patient groups stratified by clinical outcomes (smoking history and HPV status) and treatment outcomes. As shown in online supplemental figure S2, patients in the CR/PR group exhibited the highest level of total rearrangement as well as the highest diversity level (as indicated by the lowest productive Simpson Clonality). Interestingly, the SD patient group showed obviously lower number of total rearrangements than the other two groups. When comparing the Simpson Clonality among three treatment outcome groups, as expected, CR/PR patients demonstrated increased diversity (lower Simpson Clonality) than SD and PD patients, although statistically not significant (figure 3A). When comparing the two smoking groups (figure 3B), we observed significantly higher diversity (lower Simpson Clonality) in the never smoked patients with HNSCC (Wilcoxon, p=0.015); and the pattern remained when patients were further stratified by their HPV status (figure 3C). Similar to what was previously reported,^11^ HPV^+^ and HPV^–^ patients showed similar TCR diversity in HNSCC PBMC samples. We next explored the TCR diversity discrepancy by comparing the Hill number values across five order numbers (Q values). As shown in the online supplemental figure S2B–D, the CR/PR only showed a margin association with higher diversity metrics. However, the trend toward higher diversity became more evident when we compare Hill number within a more homogenous clinical subgroup of patients with a smoking history and within a subgroup of patients with HPV-negative patients only.

In addition to the analysis of the TCR repertoires in baseline samples, we also tracked the changes of TCR diversity across different phases after the treatment. For each patient, an average of ~3 time points (including the baseline) were examined (online supplemental figure S3). When comparing the diversity curves in the CR/PR group and PD group based on either the total arrangement or the Simpson Clonality score, no notable and consistent changing pattern was observed. The overall TCR diversity curves in the CR/PR group remained stable in the first two time points but showed a trend toward decreased TCR diversity (represented by decreased total arrangements) towards the end of treatment (around C4D1) in multiple patients, suggesting a potential key time point for future patient monitoring.

### Global TRBJ gene usage pattern as a potential biomarker for treatment outcome in HNSCC

In an exploratory analysis of the variable (V) and joining (J) gene usage patterns across CR/PR, SD and PD groups, we found no significant association between TRBV genes (from baseline PBMC samples) and the treatment outcomes. The CDR3 spectratyping plots (online supplemental figure S4) revealed that genes such as TRBV12-1 and TRBV19-1 are the most frequently utilized gene segments across all CDR3 sequence lengths, and the majority of the oligoclonal TRBV genes appear to be in the 13–15 amino acid-long CDR3 sequences.

**Figure 4:**
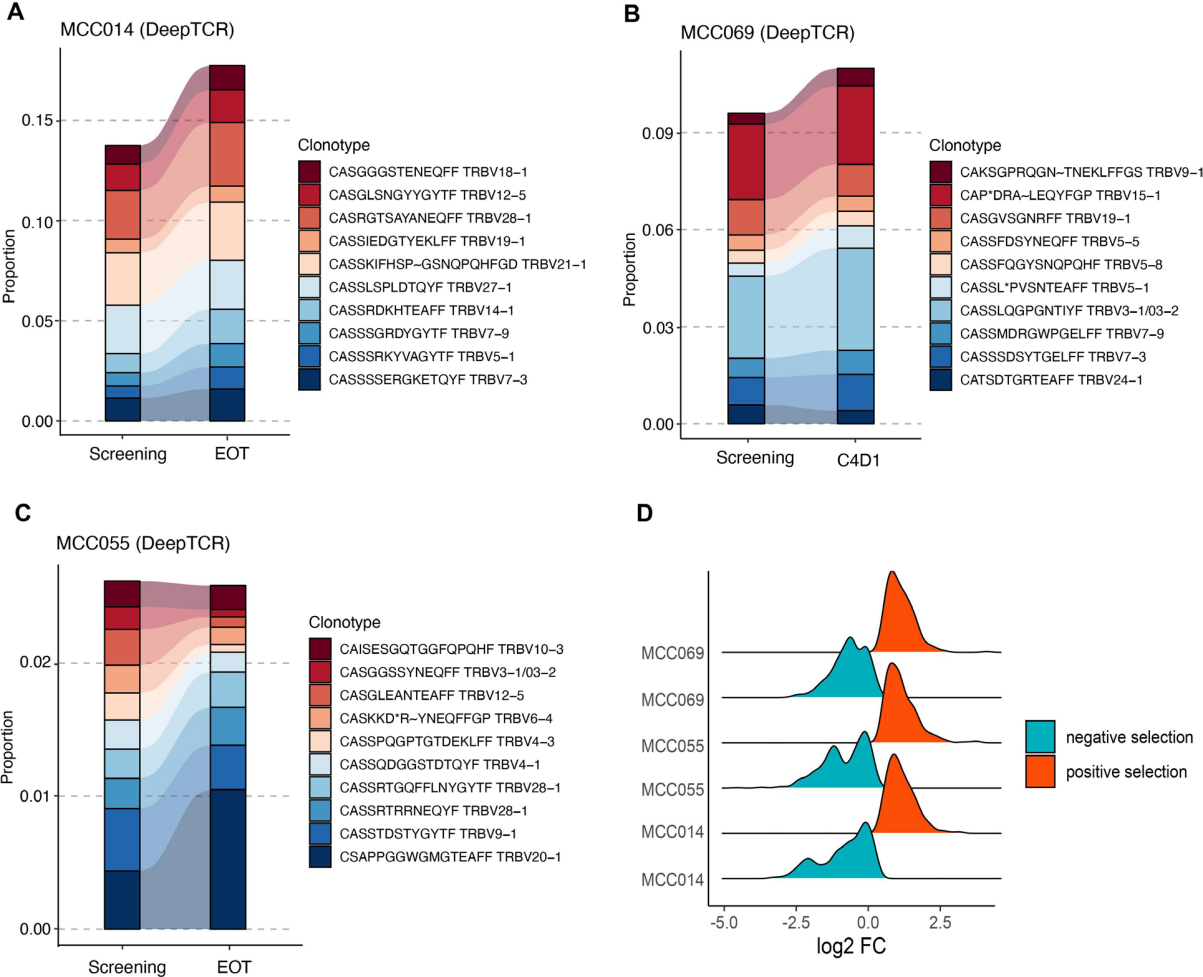
The combination therapy induces clonotype modulation in CR patients, represented by the top clonotype profiles. (A–C) Tracking the relative abundance (estimated from DeepTCR) of top 10 most abundant clonotypes in three patients in CR: (A) MCC014(A), (B) MCC069, and (C) MCC055. (D) The density distribution of TCR aa sequences under positive and negative selection after the combination therapy. The log2FC (fold-change) is calculated by log2 transformation of clonotype (relative) frequency fold-change between EOT and pretreatment time points. CR, complete response; EOT, end of treatment; TCR, T cell receptor.

Surprisingly, the abundance of TRBJ genes revealed a distinguishable pattern in patients between CR/PR and PD, which could be used as a predictor of treatment outcome. As illustrated in gene usage distribution plots (online supplemental figure S5A, B), the proportion of TRBJ2-7 dominated the repertoires of patients with CR/PR (varying between 15% and 20% in the majority of patients), followed by gene TRBJ2-1 and TRBJ2-3. However, in the PD patient group, while overall TRBJ2-7 remained as the most frequent gene, the overall usage spectrum had a more uniform distribution across multiple genes such as TRBJ1-1, TRUBJ1-2, TRBJ2-1, TRBJ2-3 and TRBJ2-5 (with the gene usage proportion close to 10% in most cases). Using the multidimensional scaling analysis and two unsupervised clustering methods, the K-means and a hierarchical clustering (online supplemental figure S5C–E), we identified two major clusters that were dominated by CR/PR patients or PD patients. Although the sample from patient MCC069 (CR) was incorrectly clustered with PD patients, it was distant from other points in the cluster. The alternative density-based spatial clustering approach identified a single cluster of eight patients who are all from the PD group, suggesting that TRBJ can be developed as a high-specificity marker for predicting PD outcomes.

**Figure 5:**
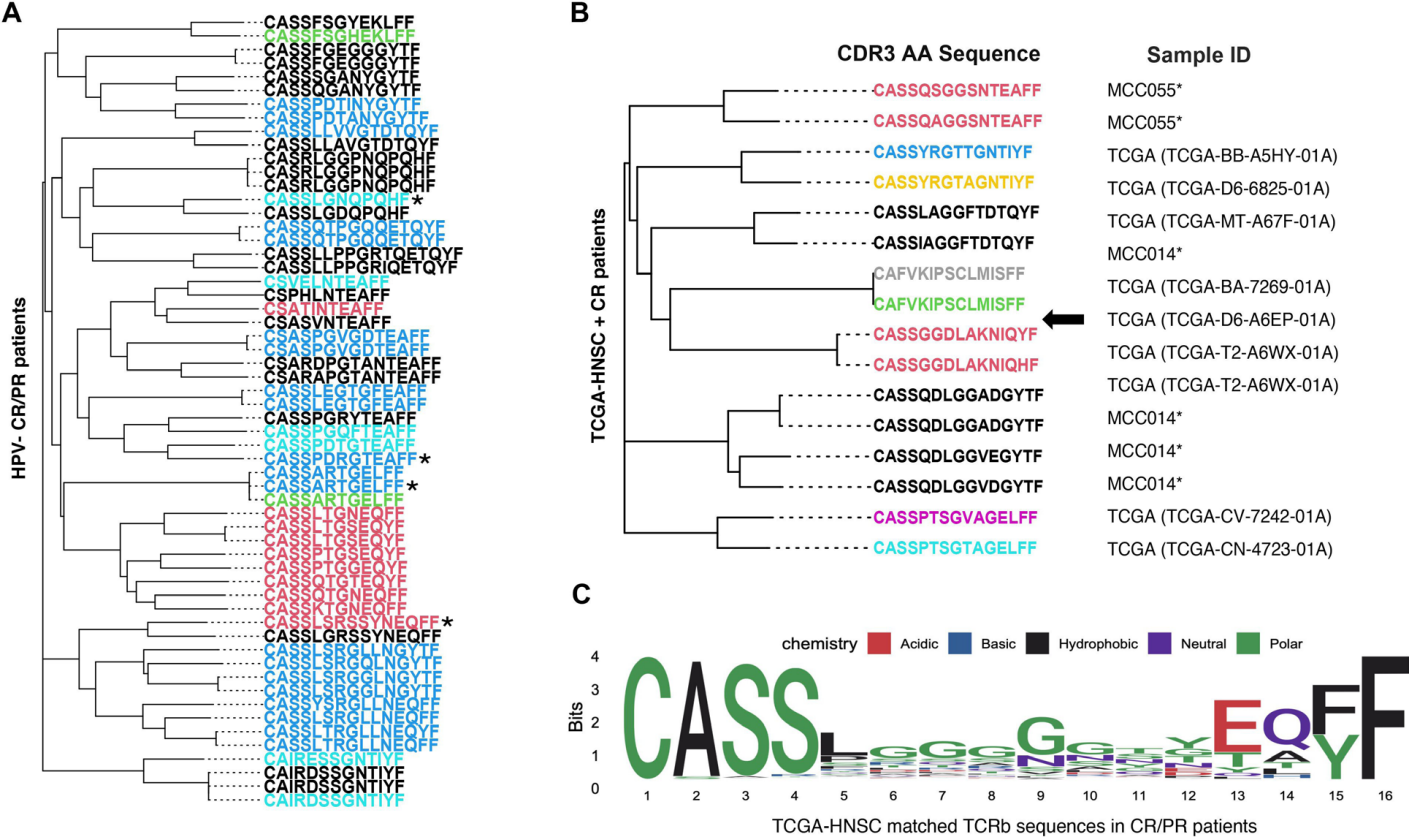
The GIANA clustering analysis revealed shared clonotypes across responder patients. (A) The clonotype clustering analysis of all HPV-negative responders (CR/PR) based on the distance calculated from the software GIANA. Sequences of special interest are marked with an asterisk (*). (B) The combined GIANA clustering reveals shared clones between peripheral TCR repertoires of CR patients and tumor samples from TCGA-HNSC. (C) The sequence logo plot shows the amino acid pattern of all CDR3 reads in CP/PR patients matched to the sequences retrieved from TCGA-HNSC. CDR3, complementarity-determining region 3; CR, complete response; PR, partial response; TCR, T cell receptor.

### Tracking shared clonotypes between time points and across patients

For each patient that received CR or PR, we first tracked the most prevalent TCR clonotypes in the PBMC samples collected at before and after the combination therapy. To ensure a more reliable abundance estimation across the entire spectrum of clonotypes, we focused on analyzing CDR3 sequence data generated from the DeepTCR assay. As illustrated in figure 4A, B, the total proportion of the top 10 most abundant amino acid clonotype sequences increased in two CR patients (MCC014 and MCC069). The other CR patient (MCC055) also showed increased relative abundance in the top sequences, with a drastic expansion in the top one sequence (figure 4C). We reason that one possible cause for this pattern is that the peripheral clonotype kinetics reflect the final effects of tumor elimination, which results in a global decrease of tumor-emigrant clonotypes in the peripheral blood and, as a result, an indirect rise of pre-existing top clonotypes. Within the CR patient group, we discovered that MCC014 contained more sequences under negative selection than the other two patients (figure 1D), which offers a possible explanation for its greatest increase in the dominating sequences. We observed two distinct clonal evolution patterns within the PR patient group, as shown in online supplemental figure S6. The expansion of top sequences was observed in polyclonal PR patients (02–048, MCC016, and MCC026). Significant reductions in top CDR3 sequences were, however, noted in two oligoclonal PR patients (MCC013 and MCC035), indicating that the combination therapy might induce different selection effects on the peripheral TCR profiles.

Using the software GIANA, we performed a TCR clustering analysis based on all CDR3 sequences from patients with CR or PR. To alleviate patient heterogeneity, we only included six patients with HPV-negative HNSCC in the analysis. We used the neighbor joining tree (figure 5A) to visualize the relationship between TCR clusters across these patients (with each color indicating different patient), which was constructed based on the isometric embedding matrix calculated by GIANA. CASSARTGELFF, CASSPDRGTEAFF, CASSLSRSSYNEQFF, and CASSLGNQPQHF were identified as four cross-patient clusters. By querying the McPAS-TCR database and setting the maximum Levenshtein distance (the edit distance between two sequences) at 1, we discovered that the first two had a similar sequence derived from PBMC samples of patients with cancer^18 19^ and that the sequence CASSLGNQPQHF was previously reported in breast and colon cancer CD8^+^ TIL cells.^20 21^ Next, we performed the cross-sample clustering analysis based on the reference library generated using the tumor RNAseq data in TCGA-HNSC. Specifically, we pooled all identified TCR CDR3 sequences from tumor samples having more than three detectable clones by MiXCR (484 out of 502 samples). A total of 13,032 clones (and 9900 unique clones) were retrieved from the TCGA cohort and included in the final composite library. By performing GIANA clustering analysis of three CR patients against the composite library, we identified multiple shared reads across patient cohorts as exemplified in figure 5B (full GIANA results listed in online supplemental table S1). We further grouped all CDR3 reads identified in CR/PR patients that were also detected in the composite library. The shared pattern of the matched sequences, visualized via the sequence logo plot (figure 5C), indicates the great diversity in amino acid 5–12. Interestingly, the sequence CASSARTGELFF identified in figure 5A showed up in two TCGA tumor samples (TCGA-BA-5555–01A and TCGA-UF-A7JO-01A). At the same time, the sequence CASSLSRSSYNEQFF also yielded two matches by querying the composite library with Levenshtein distance of 1. Together, these findings point to the possibility that these shared peripheral TCR clonotypes are from tumor-resident T cell clones, or tumor-emigrant PBMCs.^10^

## Discussion

There is a lack of research on the TCR repertoire dynamics of peripheral blood in patients with head and neck cancer following immunotherapy and their relationship to therapeutic efficacy. Here, we reported immunosequencing of TCR-B chains from the PBMC obtained from patients treated with the combination of cetuximab and nivolumab for R/M HNSCC. The beta chain of the TCR is rearranged prior to the alpha chain, and each T cell clone expresses one unique beta chain in theory. With its higher specificity and diversity, beta chain is believed to interact with antigens more frequently during recognition.^22^ Additionally, T cells express beta chains considerably more abundantly than gamma and delta chains. These factors suggest that the high-throughput sequencing of TCR-B chains would be an efficient and reliable method for tracking T cell diversity and clonal expansions.

Based on the acquired sequencing profiles, we performed extensive analyses to quantify and compare the TCR dynamics in HNSCC PBMC samples, in terms of repertoire diversity, clonality, gene usage and clonotype distribution. A novel aspect of our approach is the use of patient clonotyping defined based on the proportion of large and hyperexpanded TCR clonotypes. We reason that this approach will result in more reliable and reproducible stratification of patients based on TCR repertoires in practice, because the relative abundance estimation of large clones is much less sensitive to total number of templates compared with rarer ones. Our data show that the TCR diversity and clonotyping from pretreatment samples are associated with the therapeutic response and patient survival outcome, implying that they may be used as a predictive and prognostic biomarker for immunotherapy. Overall, the results suggest that the patients in the CR or PR group tend to have increased TCR diversity as well as a higher proportion of patients with polyclonal sequences. This conclusion is consistent with what has been observed in the TCR analyses based on tumor tissues in melanoma.^4 23^ It also corroborates the finding from a blood-based TCR study in patients with HNSCC who received neoadjuvant cetuximab,^11^ in which cetuximab responders had significantly higher TCR richness in PBMC. Cetuximab alone, as an inhibitor of the epidermal growth factor receptor, has been demonstrated to boost antitumor immunity by enchaining CD8^+^ T-cell activation and dendritic cell maturation in HNSCC.^24 25^

While the overall TCR pattern in our study is notable and consistent across multiple diversity/clonality metrics, we saw a more statistically significant result when looking into the patient subgroup of HPV-negative tumors and the subgroup with smoking history, although with a smaller sample size in each stratification. The finding is consistent with our previous research,^26^ which suggests active tobacco use in HNSCC has an immunosuppressive effect by inhibiting cytotoxic T cells in tumors. In line with this result, we observed a more significant association between TCR repertoire clonotyping and OS in these patient subsets. Additionally, the multivariable Cox regression produced a more significant p value, despite the fact that the multivariable model required more df and the current sample size may be underpowered. Taken together, all these results demonstrate that the TCR composition in blood samples and its association with immunotherapy response are intrinsically subject to the substantial molecular and clinical heterogeneity found in HNSCC biopsies. The promising associations discovered from this pilot investigation call for an expanded study or new clinical trials to enroll more patients with HNSCC in each of the clinical subgroups, or even more targeted subpopulations (such as HPV-negative and ever smokers). Although it is beyond the scope of the current study, it will be of great interest to investigate whether the favorable predictive signature observed in the HPV-negative patients also applies to patients treated with other combination regimens without cetuximab in the future. One related yet more puzzling question is whether patients with HNSCC with a smoking history will have favorable or unfavorable response in monotherapies. Despite the fact that smoking has a broadly immunosuppressive effect, the increased tumor mutation burden associated with smoking is generally regarded as a positive prognostic factor.

Another interesting finding from our analysis is that the gene usage from the TRBJ genes appears to be discriminate between patients with CR/PR and PD responses. The J gene segments have been mainly ignored in the current analyses of TCR repertoires because it is significantly less diverse than the V gene segments. However, there is a paucity of conclusive studies or consensus regarding the use of TRBV gene usage as a predictive biomarker. Our data revealed no evidence of differential TRBV patterns but did indicate that the abundance of TRBJ genes tend to be more evenly distributed in the PD patient group. The unsupervised clustering analysis of all TRBJ genes was able to identify the PD patient group with high specificity, and the utility of this information should be further explored by including more responders in future studies.

Although the results presented in this study are limited to the cetuximab and nivolumab combination therapy, we anticipate that our major conclusions on pretreatment TCR repertoire diversity and clonotyping are applicable to cetuximab/nivolumab monotherapy and adjuvant therapies. The key assumption underlying this claim is that the global peripheral T cell repertoires reflect both a pre-existing immune state in host and (perhaps more importantly) the final effects necessary for tumor elimination and immune awakening, which are fundamental mechanisms in rendering a patient amenable to immune modulation through available immunotherapies. This argument is even more relevant to the HNSCC setting because metastatic/recurrent cancers often lead to stronger changes in peripheral T cell repertoires.^8^ This hypothesis was indirectly supported by the recent findings which demonstrated no distinct peripheral immune effects between anti-PD1/CTLA4 single-agent and combination therapy for treating metastatic melanoma.^10^

In summary, we acknowledge the inherent challenge of functionally interpreting specific TCR clonotype sequences discovered from PBMC samples, given the scarcity of bioinformatics databases available for cancer-specific TCR functional annotation and antigen identification. A limitation of our study is that only PBMC samples, but not the matched tumor samples, were profiled with TCR sequencing. We have applied GIANA to cluster all TCR sequences obtained from the responder group and discovered multiple shared clonotypes across individuals. More studies are needed to determine their functions and roles in antigen-specific immune modulation. Nonetheless, we believe the data and the prognostic signature presented above could impact the development of more effective blood-based markers for HNSCC treatment stratification and management.

## Data Availability

All TCGA data are available at the GDC portal (https://portal.gdc.cancer.gov). Data from ImmunoSEQ assay will be availalbe upon reasonable request.
