# Supplementary material for: T cell repertoire in peripheral blood as a potential biomarker for predicting response to concurrent cetuximab and nivolumab in head and neck squamous cell carcinoma
Source: J Immunother Cancer. 2022 Jun 8;10(6):e004512. doi: 10.1136/jitc-2022-004512 (PMC9185557; doi:10.1136/jitc-2022-004512)
Supplement: Supplementary data [file jitc-2022-004512supp001.pdf]

T cell repertoire in peripheral blood as a potential biomarker for predicting response to concurrent cetuximab and nivolumab in head and neck squamous cell carcinoma

Wang X et al. (2022)

Supplementary figures

*To whom correspondence should be addressed: Christine H. Chung, MD., Department of Head and Neck-Endocrine Oncology, Moffitt Cancer Center, 12902 Magnolia Drive, Tampa, FL. 33612.
Email: Christine.Chung@Moffitt.org

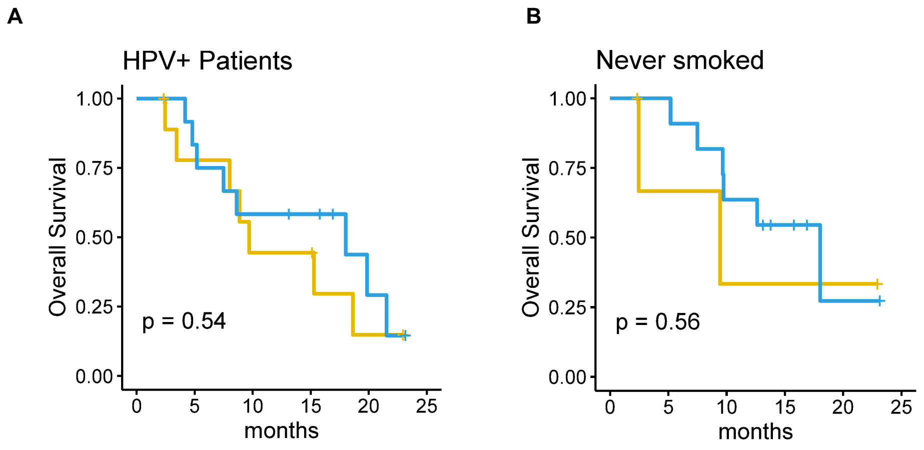

Supplementary Figure S1. Kaplan-Meier survival curves comparing patients with oligoclonal and polyclonal TCR repertories in (A) HPV-positive patients; and (B) never smokers.

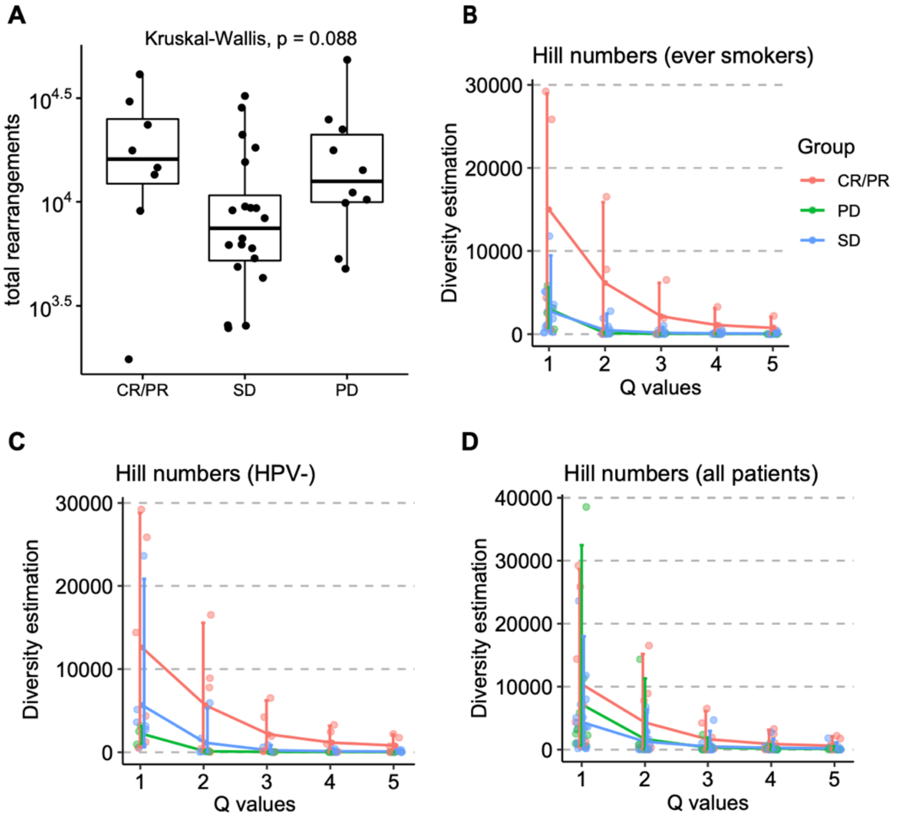

Supplementary Figure S2. Comparing additional TCR diversity measures across three treatment outcome groups. (A) The patients with CR/PR have higher total rearrangements than patients with SD or PD. (B) The responder patients who are ever smokers tend to have higher TCR diversity in baseline PBMC as represented by the Hill numbers (a combined diversity metric). (C) The responder patients exhibit higher TCR diversity in the HPV-negative patient subgroup. (D) Comparing Hill numbers of baseline PBMC from all patients across three patient outcome groups. The Q values are the order number in calculating Hill number biodiversity (Q=1, the hill number represent the Shannon entropy, Q=2 the Hill number present Inverse Simpson Index).

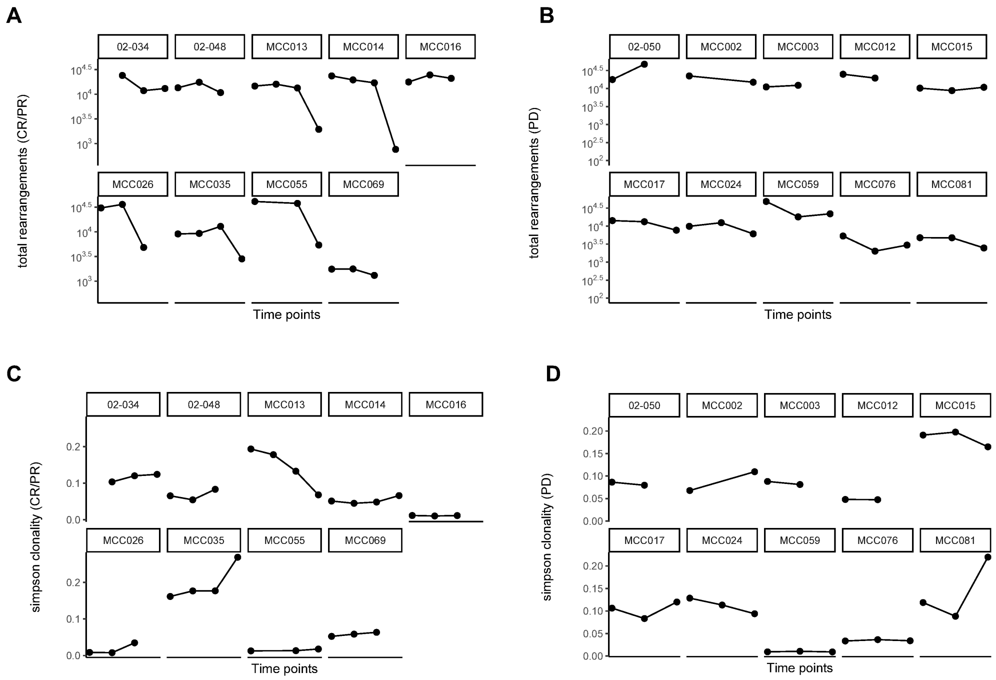

Supplementary Figure S3. Tracking and comparing the changes of TCR diversity measures (A and B: total rearrangements, C and D: Simpson clonality) across different treatment phases and treatment outcome groups (A and C: CR/PR; B and D: PD group).

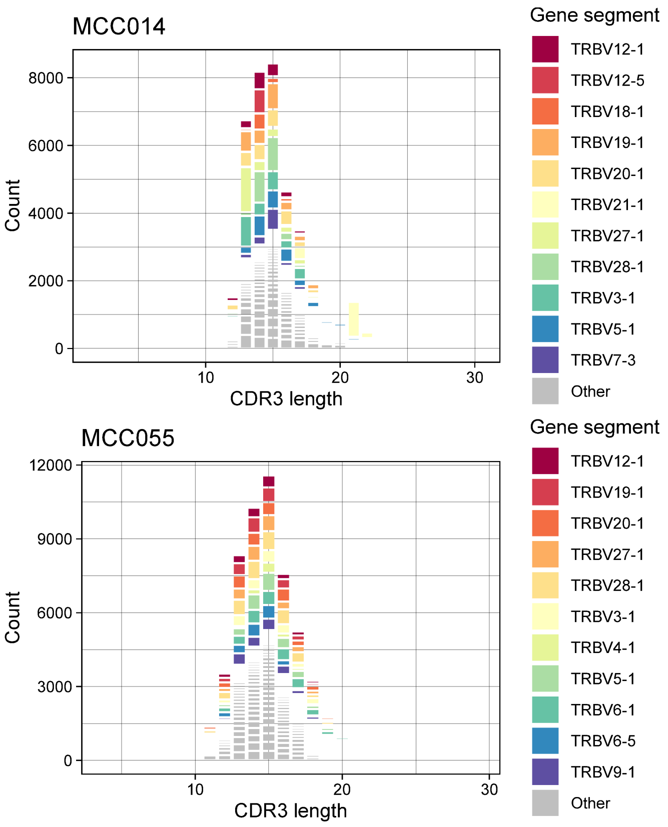

Supplementary Figure S4.
Representative CDR3 spectratyping plots (tracking gene usage across CDR3 length) from two patients in the CR group.

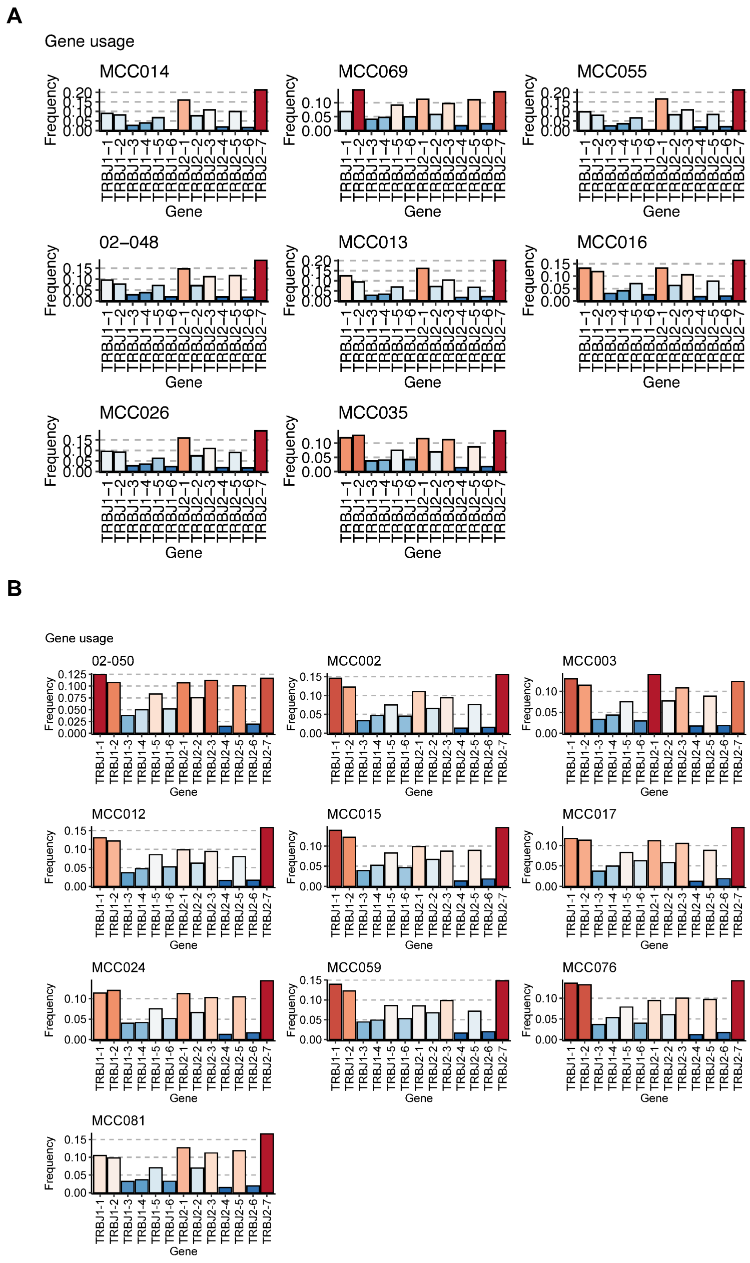

Supplementary Figure S5 (A-B). Differential TRBJ gene usage pattern by comparing CR/PR patients (A) and PD patients (B).

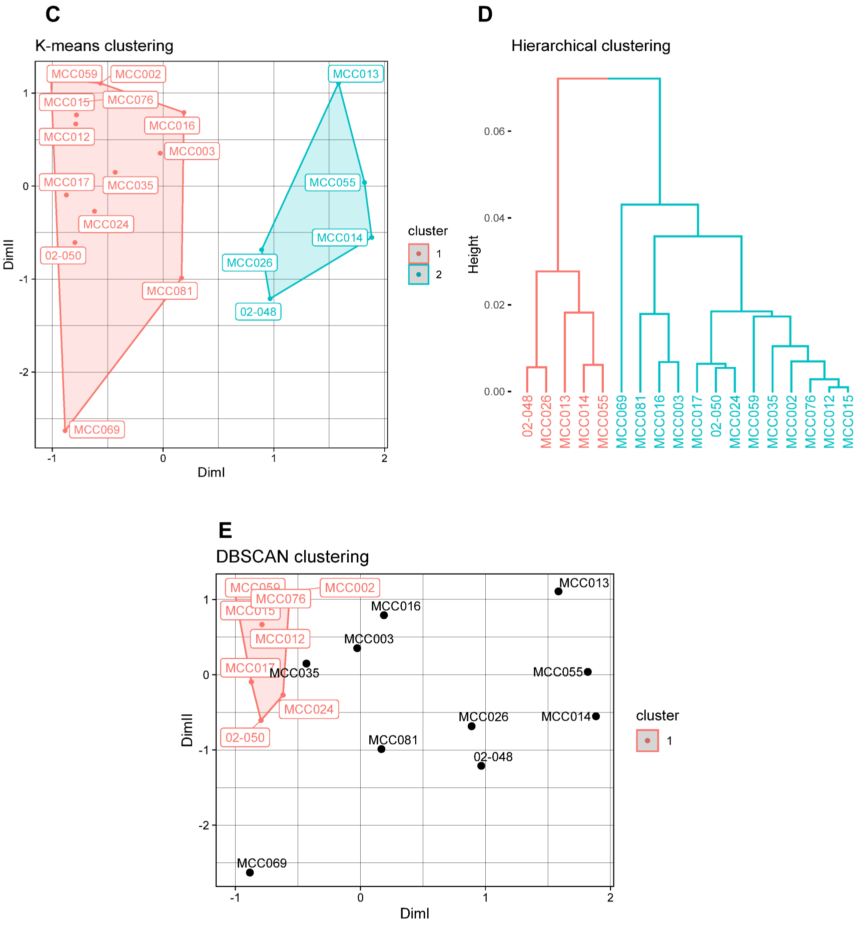

Supplementary Figure S5 (C-E). The overall TRBJ gene usage pattern is informative in distinguishing patient from CR/PR and PD groups. (A) The K-means clustering of the global TRBJ gene usage among patients identifies two clusters are predominantly CR/PR or PD patients. (B) The hierarchical clustering analysis of global TRBJ gene usage identified two major clusters similar to 3A.(C) The clustering method based on DBSCAN (The alternative density-based spatial clustering) approach identified a single cluster of eight patients who are all from the PD group.

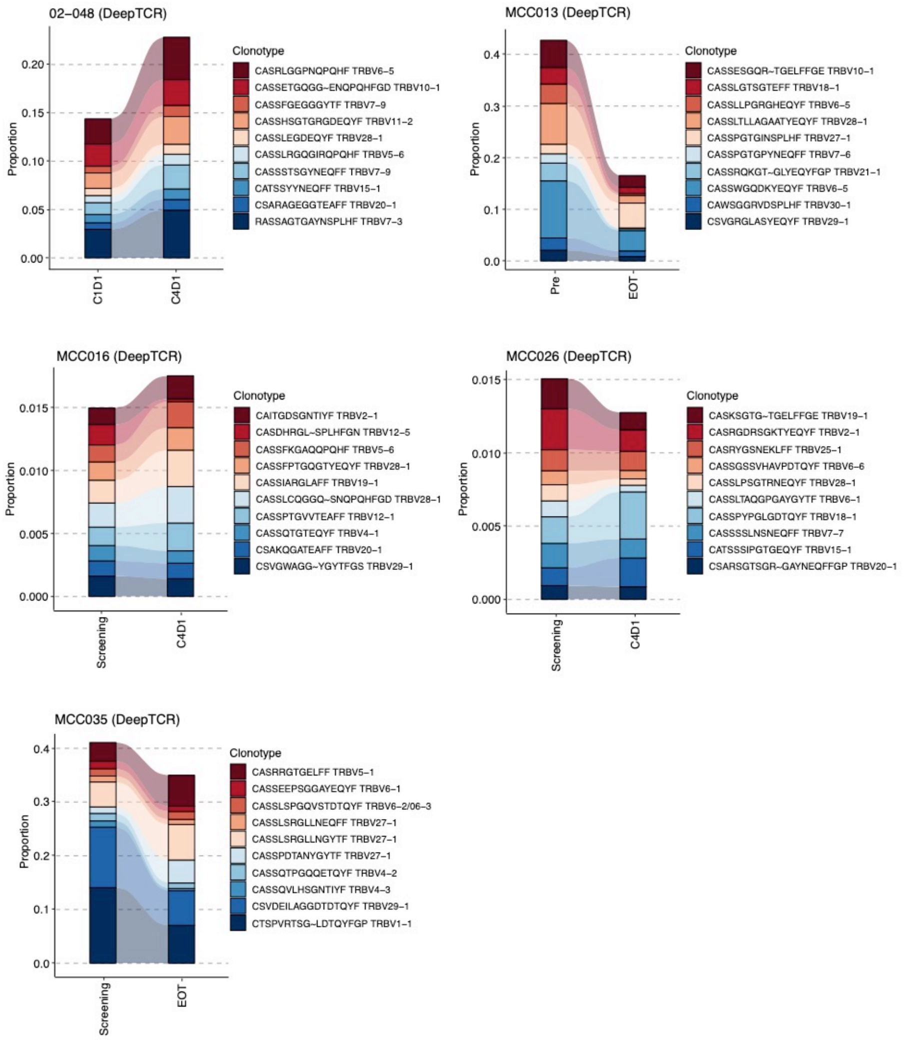

Supplementary Figure S6. Comparing the profiles of top 10 abundant TCR CDR3 sequences between pre- and post-treatment time points in five HNSCC patients with PR (based on deepTCR assay in the follow-up experiment).
